# Marital separation and contact with primary healthcare services for mental health problems: a register-based study

**DOI:** 10.1186/s40359-020-00488-0

**Published:** 2020-11-25

**Authors:** Anne Reneflot, Carine Øien-Ødegaard, Lars Johan Hauge

**Affiliations:** Mental and Physical Health, Norwegian Institute of Public Health, Nydalen, PO Box 4404, 0403 Oslo, Norway

**Keywords:** Health services, Primary healthcare, Mental health, Marital status, Marital separation, Registry data

## Abstract

**Background:**

Marital separation is associated with mental health problems, but little is known about how this translates into healthcare use. In this study, we examine the relationship between marital separation and primary healthcare use for mental health problems.

**Methods:**

We used data covering the period from 2005 to 2015 from the Norwegian Population Register, Statistics Norway’s Educational Registration System and the Norwegian Health Economics Database. Data were analyzed using logistic regression analysis. To control for time invariant characteristics, we estimated fixed-effect models.

**Results:**

Marital separation was associated with increased contact with primary healthcare services for mental health problems (MH-consultations). The prevalence of MH-consultations peaked during the year of marital separation. MH-consultations were more common following marital separation than prior to the separation. This pattern remained significant in the fixed-effect models.

**Conclusions:**

Men and women who experienced marital separation were more likely to consult primary healthcare services for mental health problems than those who remained married. Our study suggests that several mechanisms are in play. The prevalence of MH-consultations of those who eventually separated were higher several years prior to the separation. This lends support to selection mechanisms, whereas the sharp rise in the prevalence of MH-consultations around the time of marital separation coupled to higher levels several years after separation, indicate that marital separation induces both transient stress and leads to more lasting strain.

## Background

Marital separation is associated with a wide array of adverse outcomes including a deterioration in mental health [1]. Numerous studies have demonstrated that men and women who separate are more likely to report symptoms of anxiety and depression and to experience reduced life quality than those who remain married [2–9].

In contrast, only few studies have examined the relationship between marital separation and healthcare service use for mental health problems [10–12]. Studies focusing on contact with primary healthcare services are particularly sparse. All people registered living in Norway are entitled to a GP, and a consultation with a GP is only subject to a small fee-for-service. General practitioners (GPs) play an important role in diagnosing and managing mental health care and are often gatekeepers to accessing specialized mental health services. In order to understand how mental health problems in association with marital separation translate into healthcare service use, it is vital to study primary healthcare (PHC) use. A handful of studies have examined PHC use in relation to marital separation [13–15]. Based on cross-sectional data from ‘Divorce in Flanders’, Buffel et al. [15] examined self-reported contact with mental healthcare services (including GP use) among married and divorced men and women. According to their study, a higher percentage of divorced men and women were in contact with their GP for mental health problems than married men and women.

A positive association between marital separation and contact with PHC for mental health problems does not necessarily imply a causal relationship. Three explanations have been proposed for the negative association observed between divorce and mental health [7]. Both social role theory and crisis theory assume a causal relationship. While social role theory assumes being divorced is inherently more stressful than being married, stress theory attributes the negative effect of divorce to stressful role transitions and transient stressors. Social selection theory, on the other hand, argues that people with mental health problems are less likely to get married and to get divorced than people without such problems.

In order to delineate the mechanisms in play, panel data with repeated measures on PHC use before and after divorce is necessary. The process leading up to marital dissolution may start years before the actual separation, leaving a potential imprint on the involved partners’ mental health. In order to take selection into account, information about PHC use for mental health problems 5–10 years pre-divorce is considered preferable [7]. In the same line of reasoning, to examine whether a potential effect of marital separation is transient or more persistent, information several years subsequent to divorce is required. For example, a Finnish study that examined the use of psychotropic medication in association with marital separation reported excessive use of psychotropic medicine several years prior to separation, peaking 6–9 months prior to separation and stabilizing at a higher level following separation [16]. Similar patterns have been observed in studies examining the relationship between marital transitions and self-reported measures of mental health [4–6] and quality of life [6, 8].

In general, studies examining the relationship between marital separation and various mental health outcomes differ with regard to the length of the observation period, the number of waves included and sample size, and there is still a lack of high-quality panel studies [4, 6, 17]. With regard to studies of PHC use, the current literature is based on cross-sectional data and self-reported measures [13–15]. Hence, these studies are unable to conclude whether PHC use due to mental health problems increased as a result of the separation or if the prevalence of contact was high even before the separation, as well as if the effect of marital separation was more transient or long lasting. Moreover, self-reported PHC use is also likely to be plagued by recollection bias.

In this study we use Norwegian register data covering all adults from 2006 through 2015 to examine the relationship between marital separation and PHC use for mental health problems. Information about marital dissolution is linked with annually updated information about the use of primary healthcare services for mental health problems. In order to account for stable unobserved individual characteristics, we apply a fixed-effect regression analysis. Men and women are analyzed separately.

## Methods

The analysis is based on data covering the period from 2005 to 2015 from the Norwegian Population Register, Statistics Norway’s Educational Registration System and the Norwegian Health Economics Database. By means of unique personal identification numbers assigned to all Norwegian residents, it is possible to construct individual record linkages between the different data sources. The data include 1,247,700 men and women who were married by January 1st 2005, and who were still alive at the end of the observation period. The sample were followed annually until 2015. In our sample, 428,700 married men and women separated during the observation period. The analysis is restricted to men and women born in Norway between 1945 and 1984. The reason for this selection is that vital information such as education is missing for a substantial number of those born outside Norway.

There are obvious advantages to using register data. The large number of cases makes it possible to study very small groups of individuals. Further, unlike retrospective surveys, register data are not hampered by underreporting or attrition and sample selection bias [18].

### Measures

#### Outcome variable

Contact with PHC for mental health problems, a MH-consultation, was measured annually from 2006 to 2015. It is based on whether a patient had received a psychological diagnosis during a consultation with a general practitioner or a primary care emergency unit. Diagnoses are based on the International Classification of Primary Care (ICPC-2) [19]. Consultations involving any diagnosis from P01–P99 were classified as MH-consultations. Each patient may have several MH-consultations during the course of a year. Classification was by calendar year with no MH-consultations in a given calendar year classified as 0 and one or more consultations classified as 1.

#### Key independent variable

The key independent variable was marital status. The main distinction was between those who remained married throughout the observation period and those who separated. Since we wanted to estimate the immediate effect of marital dissolution on MH-consultations, we used marital separation instead of divorce. People who experience marital separation may remain separated for several years before finalizing their divorce. Therefore, the date on divorce is an unsuitable indicator for union dissolution. Those who experienced marital separation in the observation period were assigned the value 1, while those who remained married were assigned the value 0. In our data, we were not able to include dissolution in cohabiting relationships, thus only individuals married at the start of follow-up were included.

### Covariates

In addition we included control variables known to be associated both with the risk of marital separation and mental health problems such as education, age and having children [20, 21]. The following control variables were entered into the analysis: indicators reflecting time before and after marital separation, age, year, whether the respondent had children still living in the family home, and educational attainment. The time before and after marital separation was coded as dummy variables, one dummy for each year. In the analysis, we distinguished between the likelihood of taking primary education, secondary education, tertiary lower education (at least one examination at a college or university) and tertiary higher education (master’s degree or PhD). Information about whether the respondents had children living with them and the respondents’ educational status were entered as time-varying covariates. Since healthcare use may change as a function of time and age, we included both variables in the analyses. Age was entered in the analyses as a time-varying covariate grouped into the five categories less than 30 years, 30–44 years, 45–59 years, and 60 years and older.

### Ethical approval

The Regional committee for Medical and Health Research Ethics granted approval for the research project, and all registry owners consented to their data being utilized.

### Analysis

Since our outcome is a dichotomous variable, we utilized logistic regression analyses. The main objective was to examine changes in the annual prevalence of MH-consultations in association with marital separation. We used two sets of models. In the first model, we performed pooled logistic regression analysis. This is standard logistic regression analysis where we do not take into account the panel structure of our data. Since our sample includes repeated observations of the same individuals, we used robust standard errors to account for dependency in data. In this model, we compared the MH-consultation rates of those who experienced marital separation with men and women who remained married. As a second step we performed a fixed-effect analysis. Fixed-effect models are based on variation within individuals over time, taking into account all time-constant characteristics. This research design allowed us to use each individual as its own control. The advantage of this method is that all measured and unmeasured sources of bias that are constant over time are eliminated. Since those who remain married do not experience any variation in their marital status over the observation period, they were omitted from the analysis. We calculated the difference in MH-consultations before and after marital separation and averaged those differences across all individuals in the population in order to achieve an average treatment effect [22]. We ran separate models for men and women. All analyses were performed using STATA.

## Results

**Fig. 1:**
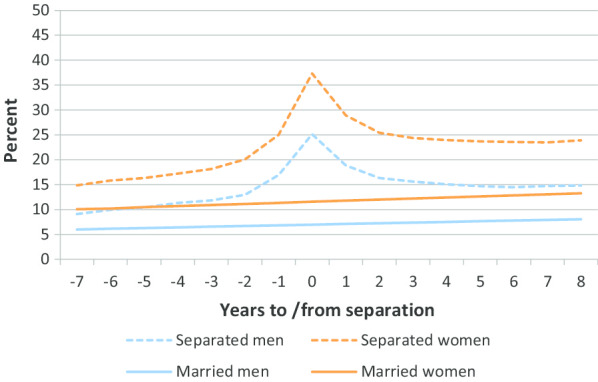
Percentage with a p-consultation with PHC in the years prior to and following marital separation by sex

Figure 1 shows the percentage with a MH-consultation in the years prior to and following marital separation. Since those married did not experience any change in their marital status, there is no before and after point to plot its prevalence of MH-consultations in relation to. Instead, we plotted the trend lines of the prevalence of MH-consultation for married men and women during the observation period (2006–2015).

Through examination, we found a remarkably similar pattern for men and women who were separated. The prevalence of MH-consultations increased steeply two years prior to separation, with a peak in the year of separation, followed by a steep decline in the two years following separation. For both sexes the prevalence of MH-consultations stabilized at a higher level after separation than prior to the separation.

For women the prevalence of MH-consultations increased from around 15% seven years prior to separation, to 37% in the year of separation. Eight years after marital separation the prevalence of MH-consultations stabilized at 24%. For men the prevalence of MH-consultations increased from 9% seven years prior to separation, with a peak at 25% in the year of separation. Eight years following marital separation the prevalence stabilized at 15%.

Compared with the men and women who remain married, those who separated were more likely to have a MH-consultation both before and after separation. Seven years prior to separation the difference was close to five percentage points for both men and women, while eight years following separation the difference increased to eight and thirteen percentage points for men and women, respectively.

We observed a slight increase in the prevalence of MH-consultations among married women and men during the observation period.

### Pooled logistic regression analysis

**Fig. 2:**
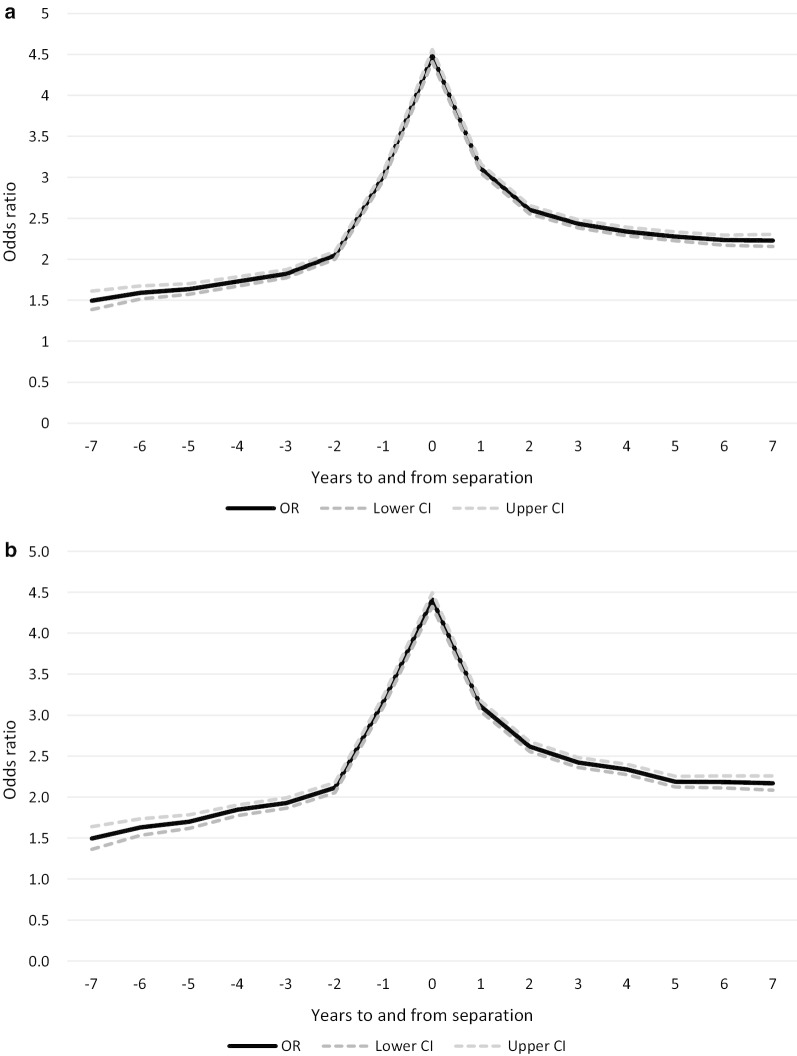
**a** Pooled logistic regression analysis. The odds of a MH-consultation (OR with 95% confidence interval) based on time to/since marital separation, women (reference group married women). **b** Pooled logistic regression analysis. The odds of a MH-consultation (OR with 95% confidence interval) based on time to/since marital separation, men (reference group married men)

Figure 2a, b show the odds of a MH-consultation for each year prior to and following marital separation, adjusted for the included covariates for men and women, respectively (see Additional file 1: table 1 for the full models). The reference group were those who remained married during the observation period. The odds of a MH-consultation follow a pattern similar to the one shown in Fig. 1. Men and women who separated had significantly higher odds of a MH-consultation both several years prior to and following separation than those who were married. Seven years prior to separation the odds of a MH-consultation were 1.5 times higher in the separated group compared to the married group. The odds of MH-consultations increased steeply two years prior to separation with a peak in the year of separation, followed by a steep decline in the two years following separation. In the year of separation, the odds of a MH-consultation were more than four times higher among the separated men and women than those who were married. Seven years following separation the odds were still more than two times higher in the separated group than in those who remained married.

Both education and living with children are associated with the risk of a MH-consultation (see Additional file 1: table 1). Having more than primary education is associated with a decreased risk of a MH-consultation, while those who live with children have a higher risk of a MH-consultation than those who do not live with children. The risk of a MH-consultation increases slightly over the study period. Among women, the risk of a MH-consultation is highest in the age groups 30–44 years and 45–59 years, while for men the risk of a MH-consultation decreases with age.

### Fixed-effect analysis

**Fig. 3:**
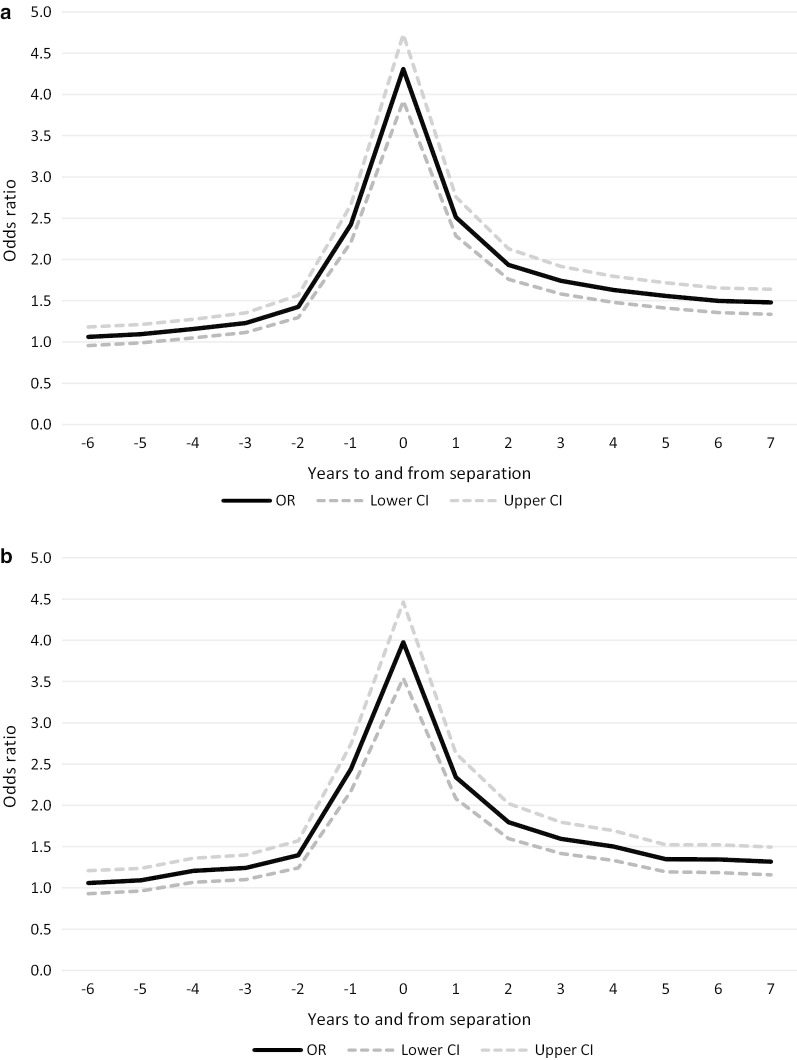
**a** Fixed-effect regression analysis. The odds of a MH-consultation (OR with 95% confidence interval) based on years to/since marital separation, women (reference group 7 years prior to marital separation). **b** Fixed-effect regression analysis. The odds of a MH-consultation (OR with 95% confidence interval) based on years to/since marital separation, men (reference group 7 years prior to marital separation)

Figure 3a, b show how the odds of a MH-consultation vary with time up to and after marital separation in the fixed-effect analysis for men and women, respectively (see Additional file 1: table 2 for the full models). The reference point in time was seven years prior to marital separation. When we accounted for all time-invariant characteristics, the time pattern of MH-consultations was still remarkably similar to the patterns shown in Fig. 1 and in the pooled logistic regression analysis (Fig. 2a, b). The odds of a MH-consultation increased in the years leading up to marital separation. The increase was particularly steep in the two years leading up to the separation. In the final year prior to marital separation, the odds of a MH-consultation were around 2.5 times higher than seven years prior to the separation. In the year of separation, the odds of a MH-consultation were around 4 times higher. Following marital separation, the odds of a MH-consultation fell, but remained at a significantly higher level than seven years prior to separation. This pattern was evident for both men and women.

## Discussion

In this study we examined the relationship between marital separation and contact with PHC services for mental health problems using high quality panel data. Compared with the men and women who remain married, those who eventually separated were significantly more likely to have a MH-consultation both several years prior to separation and several years after separation. In the year of separation, one in four men and more than one in three women contacted PHC services for mental health problems. Although the odds of a MH-consultation decreased substantially following the years of marital separation, it remained at a higher level than before the separation. This is in line with studies that have examined symptoms of mental health problems [4–6, 10], quality of life [6, 8] and the use of psychotropics [16].

As did Buffel et al. [15], we found a positive relationship between marital separation and MH-consultation rates for both men and women. This indicates that both men and women are negatively affected by marital separation. However, the strength of this relationship may differ. To test this, we performed an interaction analysis (results not shown). The results did not provide support for an interaction effect.

This study also shed some light on the mechanisms in play. We found support for both social selection and a causal relationship between marital separation and MH-consultations. Evidence suggesting that men and women who eventually separate are more likely to be in contact with PHC services for mental health problems than the continued married, up to 7 years prior to the separation, adds support to the selection explanation. The strong increase in MH-consultations in the two-year period leading up to marital separation suggests that marital discord and conflicts in these last years prior to separation increase the risk of mental health problems. After taking time invariant characteristics into account, we still found that marital separation was associated with a strong increase in MH-consultations and that although this contact decreased with time after separation, it remained at a higher level than prior to separation. This suggests that marital separation may cause transient mental health problems, and that for some people these problems persist.

The results from our study indicate that marital separation is a distressing event that leaves a potentially long-lasting imprint on the mental health of men and women. However, this group is also more prone to experience mental health problems several years prior to separating. Prevention and adequate treatment of mental health problems among adults in general may in turn potentially prevent marital separation. In order to alleviate and prevent the mental health problems accompanying marital separation, more knowledge into factors causing and maintaining such problems is required.

Future research should look more closely into the nature of the mental health problems that separated present to their GPs. It would also be important to gain more knowledge about the treatment offered by GPs. Increased knowledge regarding the extent to which those separated are offered any form of psychological therapy in the PHC services, psychotropics, referral to specialist healthcare services, or utilisation of sick leave is warranted. Finally, it is important to address how changes in life circumstances in association with marital separation (e.g. reduced household income, custody arrangements, moving) may impact the mental health of those experiencing marital dissolution.

### Strengths and limitations

To our knowledge, this is the first study to address contact with PHC services for mental health problems in relation to marital separation utilizing a prospective design, high-quality panel data with a large sample size, and a long observation period with annual updates. Previous studies have been based on cross-sectional data, which is unsuited for examining the mechanisms at play in martial dissolution.

The outcome, a consultation with the PHC services for mental health problems, was collected from a high-quality and reliable administrative register, reflecting reimbursement to GPs for consultation expenses not covered by patients’ fee-for-service. In addition to complete data, the utilization of this kind of data eliminates possible problems related to risk of self-report bias and sample attrition, as may be the case with longitudinal survey data. Other studies addressing PHC service use in relation to marital separation have been based on self-reported measures of health care use, and may as such be vulnerable to both recollection bias and sample attrition.

The main limitation of this study is that we were not able to include separation among cohabitants. In Norway and the other Nordic countries, cohabitation is considered largely indistinguishable from marriage [23, 24]. Cohabitation is the preferred first union among young adults, with more than half of all first-born children being born to cohabiting parents. In the Nordic countries in general, cohabitation enjoys wide social acceptance. On the other hand, cohabiting unions are less stable than marriage and cohabitation typically also involve more short-lived unions [25]. Hence, it is difficult to generalize our findings to separation in cohabiting unions. However, in marriage-like cohabiting unions, that is long-lived cohabiting unions and those involving children, one may find similar MH-consultation patterns in relation to separation as reported in our study. Another group not included in our study are same-sex unions. In Norway, there are about 300 same-sex marriages annually [26]. A study from 2014 found a higher divorce rate among same-sex couples than opposite-sex couples, but little is so far known about the relationship between marital separation and mental health problems in same-sex couples. Finally, we have not accounted for re-partnering among those who separated. This may have contributed to an underestimation of the more long-term effects of marital separation.

## Conclusion

Men and women who experienced marital separation were more likely to be in contact with PHC services for mental health problems than were those who remained married. Those who eventually separated had higher contact rates also several years prior to separation, whereas the sharp rise in service use around the time of marital separation, as well as higher levels of service use several year after separation, indicate that marital separation induces transient stress, but also leads to more lasting strain for some people.

## Supplementary information

**Additional file 1:** **Table 1.** Pooled regression estimates: the odds of a MH-consultation (OR with 95% confidence interval) by sex. **Table 2.** Fixed-effect estimates: the odds of a MH-consultation (OR with 95% confidence interval) by sex.

## Data Availability

The datasets generated and analysed for the current study are not publicly available due to data protection reasons.

## Abbreviations

GP: General practitioner
MH-consultation: Whether a patient had received a psychological diagnosis during a consultation with a general practitioner or an emergency unit
PHC: Primary healthcare
