# Supplementary material for: Marital separation and contact with primary healthcare services for mental health problems: a register-based study
Source: BMC Psychol. 2020 Nov 25;8:124. doi: 10.1186/s40359-020-00488-0 (PMC7687816; doi:10.1186/s40359-020-00488-0)
Supplement: Supplementary file 1 — Additional file 1: Table 1. Pooled regression estimates: the odds of a MH-consultation (OR with 95% confidence interval) by sex. Table 2. Fixed-effect estimates: the odds of a MH-consultation (OR with 95% confidence interval) by sex. [file 40359_2020_488_MOESM1_ESM.docx]

**Table 1 Pooled regression estimates: the odds of a MH-consultation (OR with 95% confidence interval) by sex**

|  | Females | | | Males | | |
| --- | --- | --- | --- | --- | --- | --- |
|  | OR | 95% CI | | OR | 95% CI | |
| **Time to/from separation** |  |  |  |  |  |  |
| Still married | 1 |  |  | 1 |  |  |
| 7 years | 1,495 | 1,388 | 1,611 | 1,494 | 1,362 | 1,640 |
| 6 years | 1,592 | 1,514 | 1,674 | 1,631 | 1,533 | 1,734 |
| 5 years | 1,637 | 1,574 | 1,704 | 1,699 | 1,619 | 1,782 |
| 4 years | 1,728 | 1,673 | 1,785 | 1,847 | 1,776 | 1,905 |
| 3 years | 1,823 | 1,773 | 1,875 | 1,927 | 1,863 | 1,992 |
| 2 years | 2,048 | 1,999 | 2,097 | 2,115 | 2,055 | 2,177 |
| 1 years | 3,026 | 2,972 | 3,082 | 3,173 | 3,106 | 3,241 |
| 0 | 4,484 | 4,409 | 4,560 | 4,409 | 4,324 | 4,495 |
| +1 years | 3,104 | 3,049 | 3,160 | 3,105 | 3,041 | 3,171 |
| +2 years | 2,604 | 2,555 | 2,654 | 2,617 | 2,558 | 2,677 |
| +3 years | 2,432 | 2,382 | 2,482 | 2,422 | 2,363 | 2,482 |
| +4 years | 2,338 | 2,287 | 2,391 | 2,336 | 2,274 | 2,399 |
| +5 years | 2,277 | 2,226 | 2,333 | 2,186 | 2,122 | 2,252 |
| +6 years | 2,234 | 2,173 | 2,296 | 2,185 | 2,112 | 2,260 |
| +7 years | 2,229 | 2,156 | 2,303 | 2,170 | 2,084 | 2,259 |
| **Education** |  |  |  |  |  |  |
| Primary school | 1 |  |  | 1 |  |  |
| High school | 0,843 | 0,833 | 0,854 | 0,753 | 0,742 | 0,764 |
| Lower tertiary education | 0,842 | 0,833 | 0,852 | 0,759 | 0,746 | 0,771 |
| Higher tertiary education | 0,630 | 0,617 | 0,643 | 0,508 | 0,496 | 0,520 |
| **Living with children** |  |  |  |  |  |  |
| No | 1 |  |  | 1 |  |  |
| Yes | 1,151 | 1,136 | 1,667 | 1,078 | 1,060 | 1,096 |
| **Year** | 1,023 | 1,022 | 1,025 | 1,024 | 1,022 | 1,025 |
| **Age** |  |  |  |  |  |  |
| 29 years and younger | 1,123 | 1,091 | 1,157 | 1,288 | 1,231 | 1,350 |
| 30-44 years | 1,262 | 1,243 | 1,281 | 1,161 | 1,140 | 1,182 |
| 45-59 years | 1,196 | 1,180 | 1,211 | 1,117 | 1,101 | 1,134 |
| 60 years+ | 1 |  |  | 1 |  |  |

**Table 2 Fixed-effect estimates: the odds of a MH-consultation (OR with 95% confidence interval) by sex**

|  | Females | | | Males | | |
| --- | --- | --- | --- | --- | --- | --- |
|  | OR | 95% CI | | OR | 95% CI | |
| **Years to/from separation** |  |  |  |  |  |  |
| 7 years | 1 |  |  | 1 |  |  |
| 6 years | 1,062 | 0,955 | 1,181 | 1,059 | 0,928 | 1,208 |
| 5 years | 1,095 | 0,990 | 1,211 | 1,091 | 0,963 | 1,235 |
| 4 years | 1,157 | 1,050 | 1,276 | 1,205 | 1,068 | 1,360 |
| 3 years | 1,229 | 1,117 | 1,353 | 1,241 | 1,102 | 1,398 |
| 2 years | 1,424 | 1,295 | 1,566 | 1,397 | 1,242 | 1,571 |
| 1 years | 2,424 | 2,207 | 2,663 | 2,439 | 2,172 | 2,740 |
| 0 | 4,306 | 3,921 | 4,729 | 3,976 | 3,540 | 4,465 |
| +1 years | 2,512 | 2,286 | 2,759 | 2,339 | 2,082 | 2,628 |
| +2 years | 1,935 | 1,759 | 2,129 | 1,797 | 1,597 | 2,022 |
| +3 years | 1,740 | 1,581 | 1,916 | 1,595 | 1,416 | 1,797 |
| +4 years | 1,630 | 1,480 | 1,796 | 1,502 | 1,332 | 1,694 |
| +5 years | 1,557 | 1,411 | 1,717 | 1,348 | 1,194 | 1,522 |
| +6 years | 1,498 | 1,356 | 1,655 | 1,345 | 1,186 | 1,521 |
| +7 years | 1,479 | 1,335 | 1,639 | 1,316 | 1,159 | 1,495 |
| **Education** |  |  |  |  |  |  |
| Primary school | 1 |  |  | 1 |  |  |
| High school | 1,068 | 1,023 | 1,115 | 1,095 | 1,015 | 1,181 |
| Lower tertiary education | 1,052 | 0,996 | 1,111 | 0,975 | 0,878 | 1,082 |
| Higher tertiary education | 0,994 | 0,917 | 1,079 | 1,035 | 0,889 | 1,203 |
| **Living with children** |  |  |  |  |  |  |
| No | 1 |  |  | 1 |  |  |
| Yes | 1,195 | 1,151 | 1,239 | 1,118 | 1,073 | 1,166 |
| **Year** | 1,030 | 1,028 | 1,031 | 1,032 | 1,030 | 1,034 |
| **Age** |  |  |  |  |  |  |
| 29 years and younger | 0,888 | 0,853 | 0,924 | 0,873 | 0,823 | 0,926 |
| 30-44 years | 1,182 | 1,154 | 1,211 | 1,025 | 0,995 | 1,056 |
| 45-59 years | 1,199 | 1,180 | 1,219 | 1,109 | 1,088 | 1,131 |
| 60 years+ | 1 |  |  | 1 |  |  |
